# TRD-Net: an efficient tomato ripeness detection network based on improved YOLO v8 for selective harvesting

**DOI:** 10.3389/fpls.2026.1748741

**Published:** 2026-01-28

**Authors:** Xiangpeng Fan, Xiujuan Chai

**Affiliations:** 1 Agricultural Information Institute, Chinese Academy of Agricultural Sciences, Beijing, China; 2 Key Laboratory of Agricultural Big Data, Ministry of Agriculture and Rural Affairs, Beijing, China

**Keywords:** deep learning, improved YOLO v8s, ripeness detection, SCRConv, SimAM attention, tomato selective harvesting

## Abstract

Fruit recognition and ripeness detection are crucial steps in selective harvesting. To better address the difficulties of fruit recognition and ripeness detection techniques in complex facility environments, a novel lightweight tomato ripeness detection network model based on an improved YOLO v8s is proposed (called TRD-Net). Here, a tomato dataset including 3,330 images from real scenarios was constructed, and an accurate lightweight tomato ripeness detection model trained on the captured images was developed. The TRD-Net model achieves efficient detection of tomatoes affected by overlapping occlusions, lighting variations, and capture angles, offering swifter detection speeds and lower computational demands. Specifically, the feature extraction module of YOLO v8s was refactored by employing spatial and channel reconstruction convolution (SCRConv) and adding the SimAM attention mechanism. The CIoU loss function was replaced by the MPDIoU loss function. The performance of the novel TRD-Net was comprehensively investigated. The proposed TRD-Net achieved an mAP@0.5 of 0.9581 with an improvement of 4.32 percentage points, and the model size decreased from 22.5 M to 17.6 M with an inference time of 8.7 ms per image. The number of model parameters and floating-point operations per second (FLOPs) decreased by 19.69% and 22.03%, respectively. Compared with state-of-the-art models, the proposed TRD-Net is notably promising for real-time tomato recognition and maturity detection. The study contributes to the establishment of a machine vision sensing system for a selective harvesting robot in a complex gardening environment.

## 1 Introduction

Tomatoes have extremely high nutritional value and unique edible health effects, and they are the second most consumed vegetable in the world (Xu et al., 2022). According to FAO statistics (https://faostat.fao.org), China has become the world’s largest tomato producer. Harvesting is a vital part of tomato production, and manual selection harvesting is labor-intensive, costly, and inefficient. Labor costs account for 35%–45% of the total cost of tomato production (Liu et al., 2020). In addition, labor shortages are severe (Liu et al., 2022) with the development of urbanization and aging of society. These problems restrict the high-quality and efficient development of the gardening industry (Rong Q. et al., 2023), which hinders the increasing requirements of consumers for high-standard tomatoes. Therefore, there is an urgent need for automated harvesting of tomatoes for industrialized tomato cultivation (Hou et al., 2021). The automated detection and assessment of tomato fruit maturity not only prevents incorrect harvesting and reduces costs (Kumar and Mohan, 2023; Zheng et al., 2024), but also enhances resource utilization (Li et al., 2024). The machine vision system of the tomato harvesting robot plays a crucial role because tomato detection and ripeness discrimination are prerequisite steps. Thus, developing a robust fruit visual detection algorithm and realizing real-time online detection of tomato ripeness are of great economic significance.

To address these challenges, many researchers have conducted extensive studies on fruit recognition and ripeness detection in recent years. In the early days, researchers detected fruits using conventional image processing (CIP) methods by extracting the color (Zhao et al., 2016a), shape, and texture features of the fruit (Gongal et al., 2015). CIP techniques based on manual feature extraction have limitations, including low accuracy, poor real-time performance, and poor anti-interference ability in complex environments (Zhao et al., 2016b). As intelligent algorithms in the development, deep learning (DL) models have significant advantages over conventional methods (Wang et al., 2022). With the rapid development of convolutional neural networks (CNNs) in DL, the end-to-end detection process and the advantage of automatic extraction of depth features have reduced many complex operational steps in CIP methods (LeCun et al., 2015). Various CNN have been successfully used for fruit target recognition (Vasconez et al., 2020) and have shown promising results. The detection performance of CNN usually improves with an increase in the amount of training sample data (Liu et al., 2020; Vasconez et al., 2020). The detection algorithms of CNN can be categorized broadly into two-stage and single-stage detectors (Diwan et al., 2023). Two-stage detection methods, such as Faster R-CNN (Gao et al., 2020) and Mask R-CNN (Jia et al., 2020), first enumerate the candidate boxes and then classify the objects. These methods exhibit high precision with low error rates but require long runtimes. Single-stage detection methods, including SSD (Yuan et al., 2020) and YOLO serial models (Lawal, 2021; Wang and He, 2021), have faster recognition speeds while maintaining the same level of precision as two-stage models. They are well-suited for real-time applications and have enjoyed great popularity for generic object detection. Koirala et al. established an efficient Mango YOLO model with an *F*1 value of 0.89 on the test set (Koirala et al., 2019). Their research proved that a single detector is faster than a two-stage detector with a similar accuracy. Liu et al. proposed a tomato detection model using an improved YOLOv3 (Liu et al., 2020). The total number of images was 966, and the highest precision reached 94.75%. Li et al. established the YOLO v5s-CQE model using the CARAFE module structure, which increased by 2.4 percentages compared with the original YOLO v5s (Li et al., 2023). The limitation was that small or blocked tomatoes may have been missed in detection, and the strong light spot on the tomato surface would cause incorrect recognition. Rong et al. also applied YOLO v5 to identify and localize tomato clusters; however, they only detected a mixture of all tomato clusters (Rong J. et al., 2023). Tomato maturity classification is a daunting challenge. To improve the accuracy and robustness of the ground-planted strawberry target detection algorithm, Du et al. selected YOLO v7 and modified it into the DSW-YOLO model with an SA attention mechanism and DCN-ELAN structure (Du et al., 2023). They also proposed a multitask YOLO-MCNN based on YOLO v5s to accomplish the tasks of fruit location, pose detection, and obstacle semantic segmentation with an inference speed of 19.9 ms (Du et al., 2024). As the occlusion became heavier, the underdetection rate increased from 4.8% to 21.8%. These studies indicate that the extreme environment of fruit growing has a profound influence on the robustness of the detection model. Hou et al. investigated a tomato cluster detection method using an improved YOLO v7 for cherry tomatoes (Hou et al., 2023), which indicated that the attention mechanism module and lightweight convolutional kernel can focus on critical information (Chen et al., 2024a). Xu et al. improved Mask R-CNN to analyze the spatial constraints and size differences of the fruit and stem of cherry tomatoes, and the class pixel accuracy of fruit segmentation achieved 93.76% (Xu et al., 2022). However, the large size and weak real-time performance of the Mask R-CNN model limit its application in mobile embedded devices. Zhang et al. adopted the YOLOX network model to detect tomato flowers and fruits, which outperformed the SSD model and Faster R-CNN models in dealing with the overlapping occlusion problem (Zhang et al., 2023). The efficiency and hardware cost requirements of visual perception algorithms significantly affect the overall performance of picking robots (Zhao et al., 2016b; Liu and Liu, 2023).

These results show that deep learning technology has been closely integrated with computer vision, and research in the field of fruit ripeness detection has gradually deepened. However, in terms of fruit detection in gardening environments, tomatoes are clustered and densely distributed. The natural environment is complex owing to the large difference in tomato growth form or orientation, serious collision and overlapping, and change in light intensity. The accuracy, robustness, and applicability of the prevailing methods still have problems with missing detection and low confidence when detecting distant and occluded targets. However, challenges remain in the accurate and real-time detection of tomatoes in complex horticultural environments. In addition, the volume and computational complexity of the current deep learning model are very large networks that perform multiple tasks simultaneously to reduce computational costs, which is crucial in robotic systems with long operation times, as artificial intelligence is still in its infancy in the field of agriculture. Owing to the performance limitations of edge equipment in fieldwork, it is difficult to ensure the real-time operation requirements of automatic tomato harvesting when deploying a model on these devices. Thus, there is still significant potential for balancing detection speed, accuracy, and model computational complexity for tomato detection in current research. Building on the aforementioned context, this study aims to establish a high-performance visual perception system for tomato-harvesting robots. Images of tomatoes were captured in a real scenario for data support. The advanced YOLO v8s model was selected as the base network architecture, and then it was refactored by three key improvements, denoted as “TRD-Net.” This study is expected to provide efficient and robust visual perception technical support for tomato-harvesting robots. The main contributions of this study are as follows:

(1) To align with the characteristics of the tomato ripening period, a medium-sized meticulously crafted tomato image dataset was constructed for fruit recognition and ripeness detection, which involved 3,330 tomato images under three different maturities.
(2) The TRD-Net model was built by introducing the SCRConv and SimAM modules to YOLO v8s to reduce redundant calculations and improve the learning effects of the representative characteristics of tomatoes in complex scenarios.
(3) The MDPIoU loss function was adopted to replace the CIoU loss function, optimize the network training process, and enhance positioning accuracy by avoiding distortion or omission of the detection frame caused by fruit overlap.
(4) In contrast to state-of-the-art (SOTA) models, the proposed TRD-Net achieved the highest accuracy and fastest detection speed, achieving the best balance of accuracy and computation cost for visual guidance during selective harvesting.

## 2 Materials and methods

### 2.1 Image data collection

**Figure 1:**
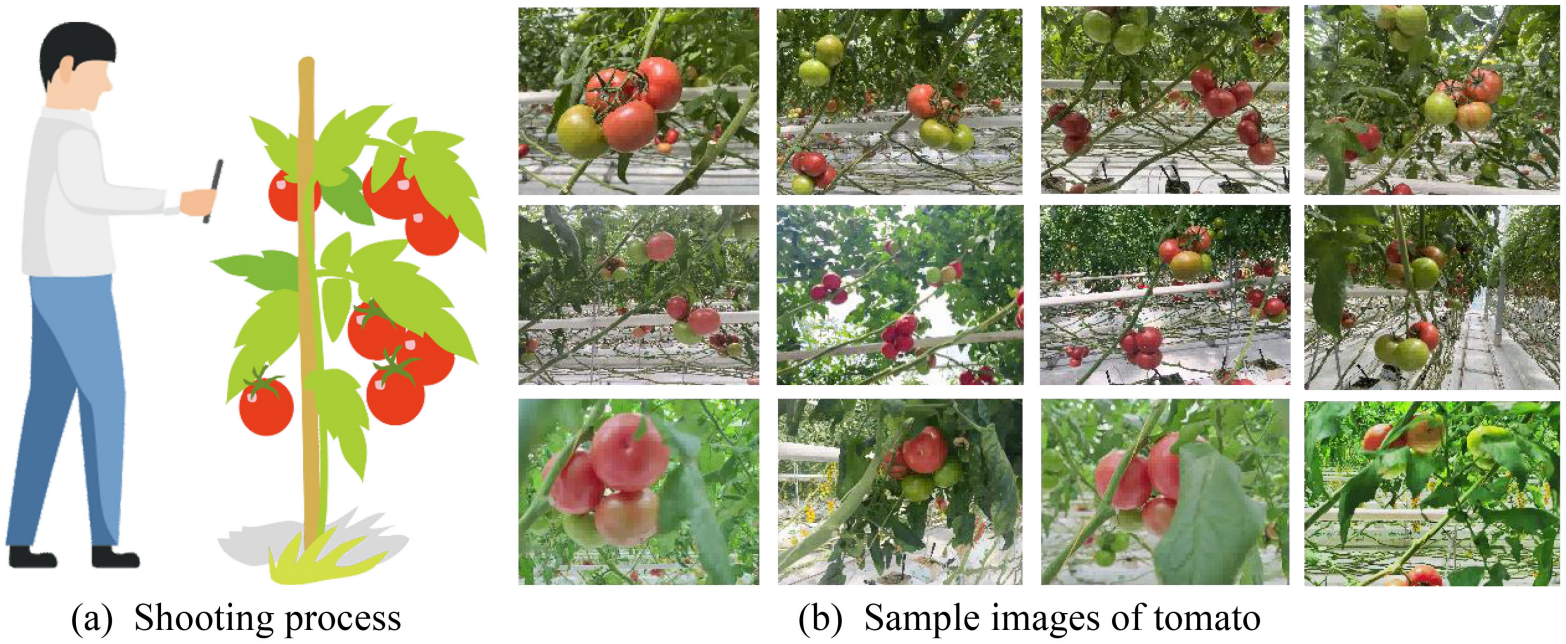
Image data collection and image samples. **(A)** Shooting process. **(B)** Sample images of tomato.

The medium-sized tomato dataset used in this study was collected during the tomato ripening period (May and June) in the smart cultivation garden of the Beijing Agricultural Vocational and Technical College in the Fangshan District of Beijing. The shooting process is illustrated in **Figure 1A**. Considering that the actual picking task requires finding ripe tomatoes at long distances and different angles, a variety of image shooting modes, such as long-distance, close-range, flat, overhead, and upward shooting, were designed. According to the natural environmental characteristics of the facility gardening, tomato images were captured under different shade levels, such as smooth light, backlight, and different shade degrees. After manually screening the different ripeness levels of tomatoes, a dataset containing 3,330 images was constructed. Examples of tomato images are shown in **Figure 1B**. From the example images, it can be observed that the texture color of the unripe tomatoes is very similar to that of the branches and leaves in the background, and different shooting angles lead to different surface brightness and occlusion degrees. The mutual shading of tomatoes and the shading of branches or leaves greatly affected identification.

### 2.2 Dataset construction and augmentation

The LabelImg annotation tool was used for the annotation process of the collected RGB image data. According to the “Industry Standard for Supply and Marketing Cooperation of the People’s Republic of China—Tomato GH/T 1193–2021,” the criteria for judging the ripeness of tomatoes can be simplified as unripe tomato, half-ripe tomato and ripe tomato. Thus, the labels included ripe, half-ripe, and unripe tomatoes, meeting the requirements of simultaneously supporting target recognition and maturity discrimination tasks for selective harvesting. The format of the saved dataset was PASCAL VOC2017, and the format of the annotation file was.xml file. The surface of unripe tomatoes is green or white-green. The surface of half-ripe tomatoes is orange-red, and the red side of the fruit is no more than 40%. The surface of the ripe tomato fruit had a red color of more than 40%. For supervised training, the open-source annotation tool LabelImg was used to manually annotate the captured tomato images, and the annotation results were saved in the PASCAL VOC format. The generated *.xml format files are saved in the pre-set folder. When the annotation process was completed, the annotation files were converted to the YOLO dataset format. After statistics, the number of ripe tomato instances was 13,922, the number of semi-ripe tomato instances was 9,473, and the number of unripe tomato instances was 12,549.

**Figure 2:**
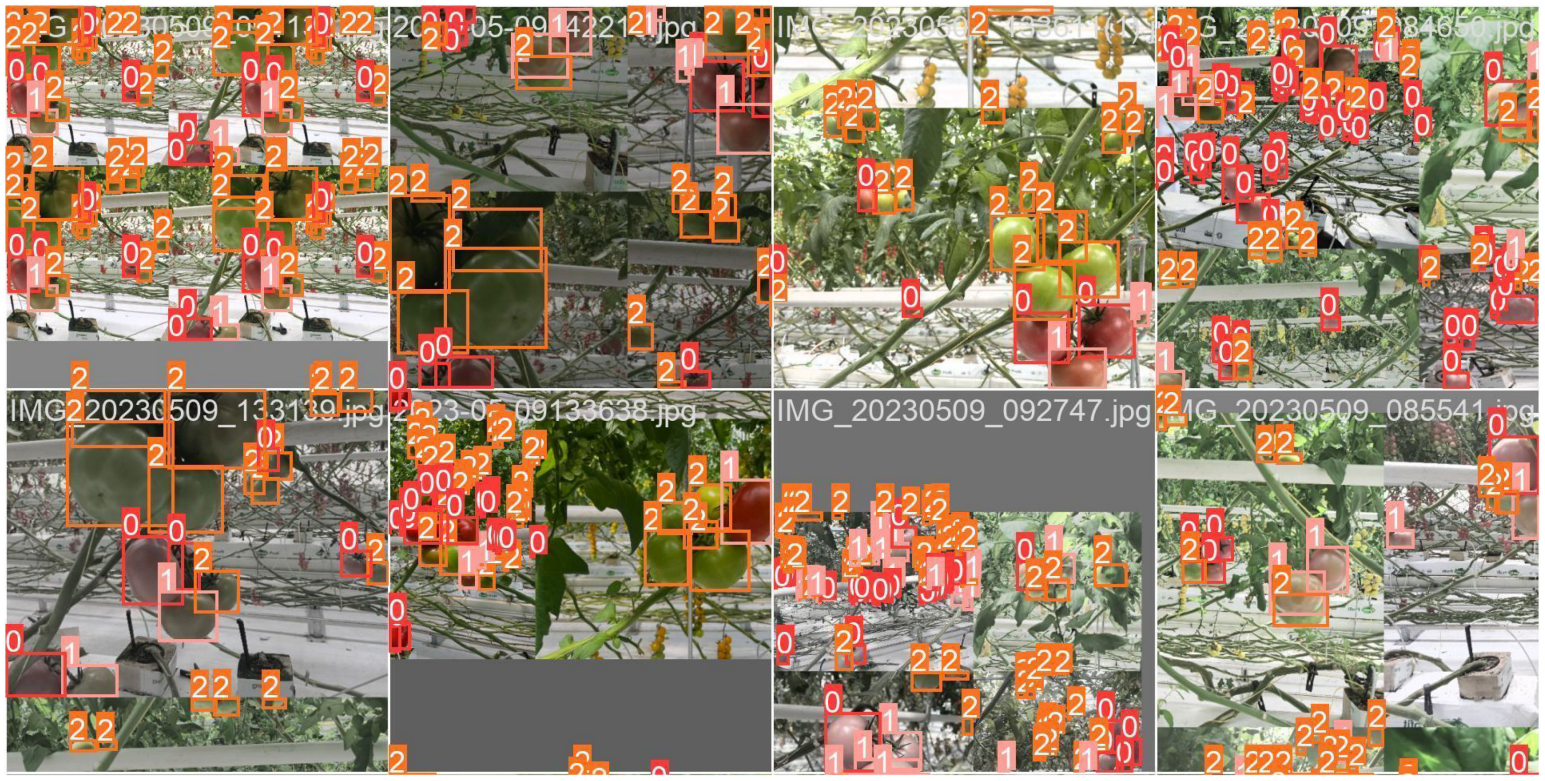
Mosaic data enhancement effects.

The dataset was then divided into training, validation, and test sets at a ratio of 7:2:1 for model training and testing. These datasets will be used for training the model and optimizing the parameters and will be compared with the predicted results to evaluate the model performance. To simulate and enhance different growth scenarios of tomatoes, such as light changes, the Mosaic data augmentation method was adopted during model training to improve the practical application effect of the training. Mosaic data augmentation involves stitching four target images into a new image according to random proportions and positions. During the training process, randomly selected image combinations simulated the diversity of complex scenes in a real scenario while retaining their respective features, making the detection model more flexible and adaptable. The results of the mosaic data augmentation are shown in **Figure 2**. This method can improve the efficiency of network training, reduce memory consumption, and boost model generalization.

### 2.3 TRD-Net model for tomato ripeness detection

#### 2.3.1 YOLO v8 and improvement

**Figure 3:**
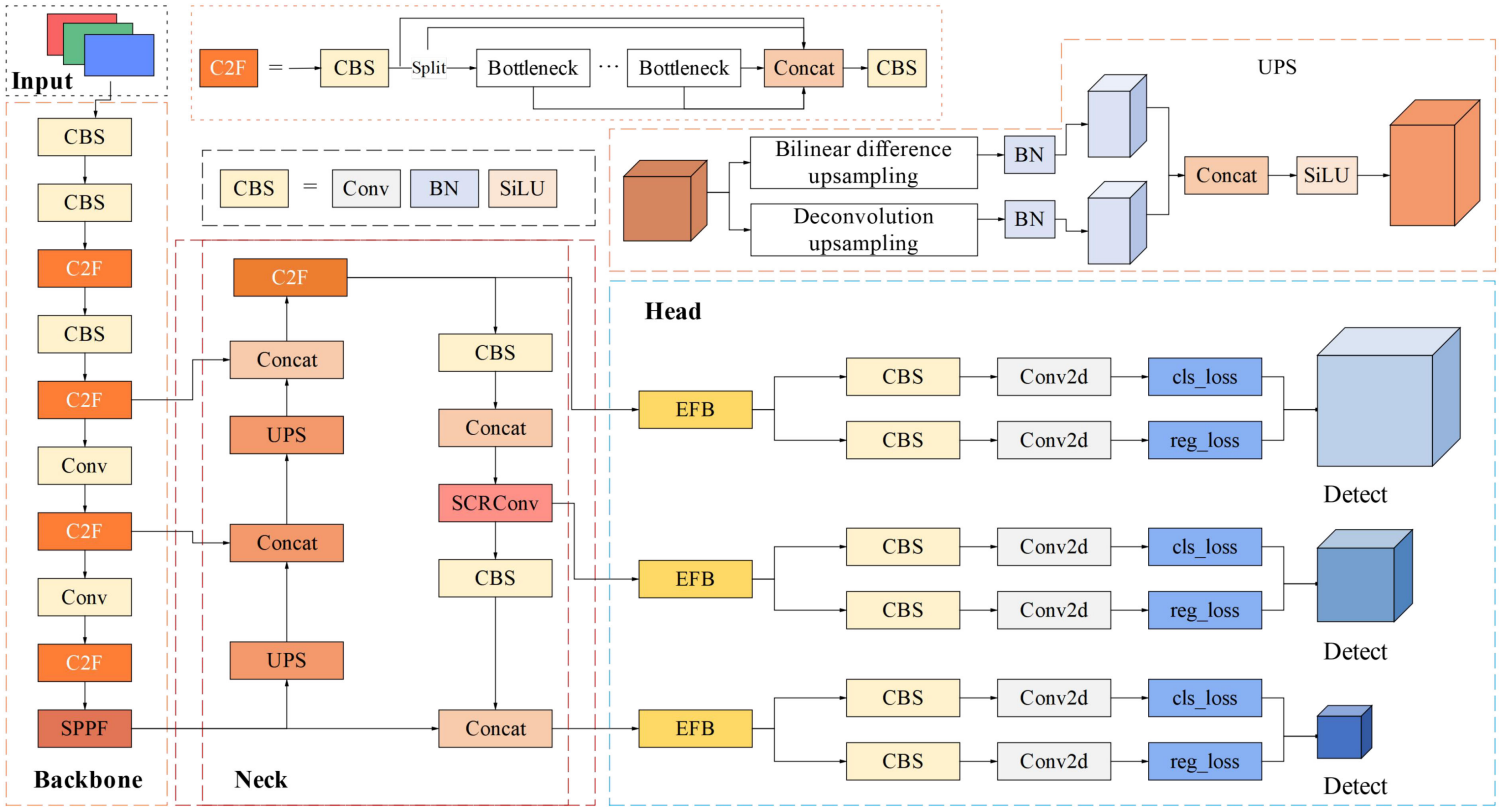
The YOLO v8s model structure.

YOLO is a real-time object detection framework designed for fast object detection and classification. It simplifies the detection task into a regression problem by converting image pixels directly into bounding box coordinates and class probabilities. YOLO v8 is a brand-new SOTA model launched by Ultralytics in January 2023 (Terven and Cordova-Esparza, 2023). It inherits the advantages of the YOLO series model but adds new features and improvements. The anchor-free mechanism (Ge et al., 2021) and lightweight framework (Zeng et al., 2023) of the YOLO serial model reduced the model operation and CPU load and accelerated the network inference. Through a comparison of the current YOLO series algorithms, it was found that the detection speed and accuracy of the YOLO v8 algorithm are superior to those of other mainstream object detection algorithms (Reis et al., 2023; Terven and Cordova-Esparza, 2023). As shown in **Figure 3**, the whole model of YOLO v8 is mainly divided into four parts: Input, Backbone, Neck and Head. The Input mainly includes mosaic data enhancement, adaptive anchor frame calculation, and adaptive grayscale filling. The Backbone network includes Conv, C2f, and spatial pyramid pooling fusion (SPPF) structures. Among them, the C2f module is the main module for learning the residual characteristics. This module is connected through multiple branches and cross-layers. The Neck network adopts a path aggregation network (PAN) structure, which can strengthen the feature fusion ability of the network for objects with different scales. The Head network decouples the classification and detection process, mainly including loss calculation, target detection box, and screening. YOLO v8 is available in five scaled versions: YOLO v8n (nano version), YOLO v8s (small version), YOLO v8m (medium version), YOLO v8l (large version), and YOLO v8x (extra-large version), where the width and depth of the convolution module vary depending on the specific application and hardware requirements. Although YOLO v8 has a much higher overall performance and flexibility than YOLO v5, it has a more abundant gradient flow, which results in a significant computational overhead while extracting more features. The visual perception task of picking robots is usually limited by equipment resources and mobile devices. Thus, the lightweight and real-time performance of the model while maintaining high accuracy is our investigation goal.

**Figure 4:**
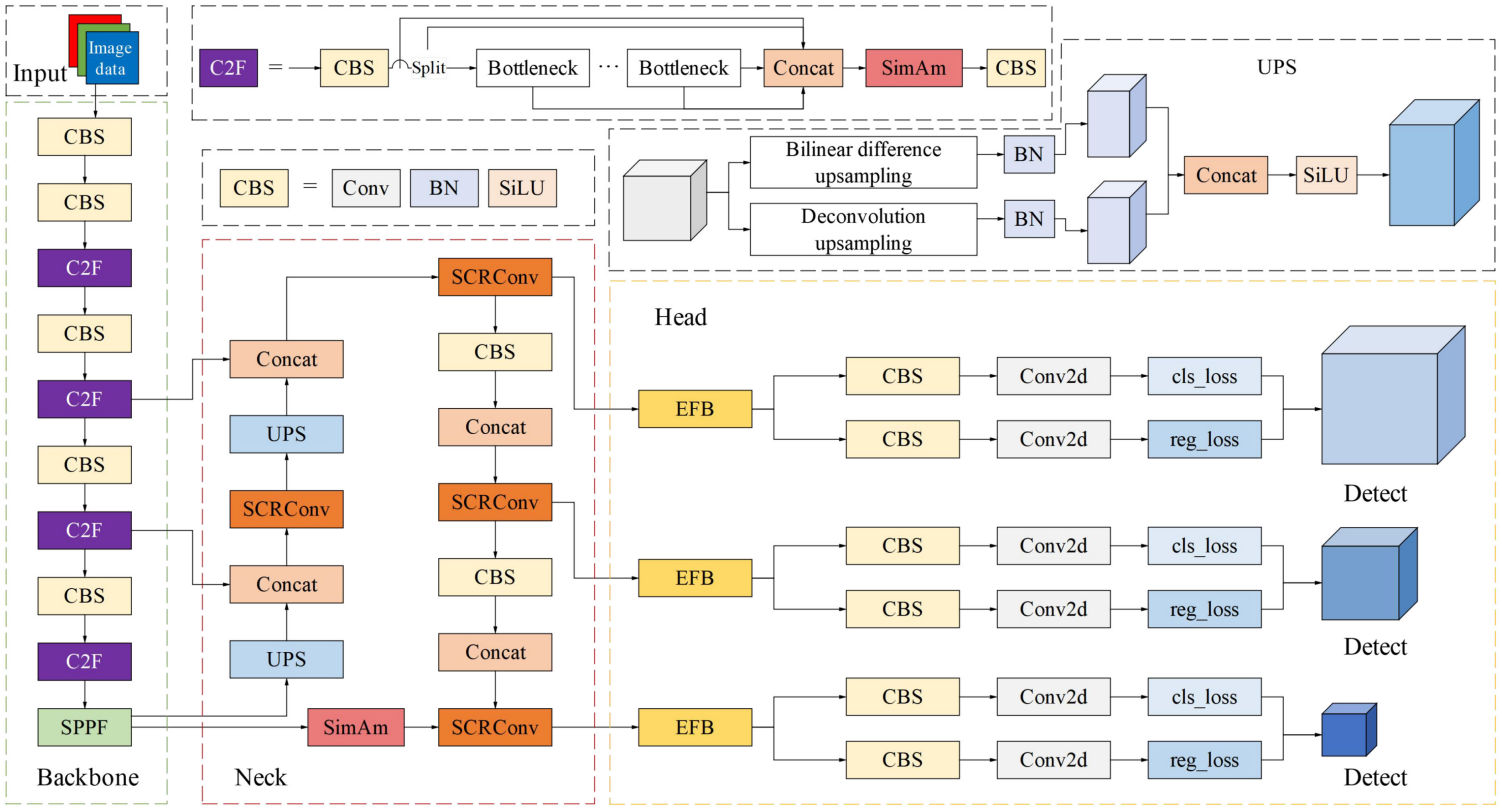
The TRD-Net model structure.

Considering the relatively fast detection speed and high accuracy, YOLO v8s was used as the base network, which can be deployed on low-cost devices such as embedded systems. After determining the goals of the model, which were lightweight, low-latency, and high precision, several improvements were made to the YOLO v8s model. Specifically, 1) SCRConv was designed by combining the spatial reconstruction convolution unit (SRCU) and channel reconstruction convolution unit (CRCU) to boost the feature representation efficiency and reduce the redundancy of the input feature map. 2) The SimAM module was introduced to make the model focus more on the key features of tomatoes in complex environments, which aims to enhance feature representation and model learning efficiency. 3) The MPDIoU loss function is utilized to replace the original CIoU loss function, which could solve the distortion of the detection frame caused by fruit overlap and effectively reduce the problem of missed detection of tomato fruits. Based on the above improvements, the final TRD-Net model structure was constructed, as shown in **Figure 4**.

#### 2.3.2 Spatial and channel reconstruction convolution module

SCRConv was designed by integrating the SRCU and CRCU to minimize the spatial and channel redundancy of features. SRCU uses a separation and reconstruction method to suppress spatial redundancy, whereas CRCU uses a split-transform-fusion strategy to reduce channel redundancy. Specifically, the SCRConv principle is to first obtain the spatially refined feature *X^W^* through the SRCU and then obtain the channel refinement feature *Y* through the CRCU.

**Figure 5:**
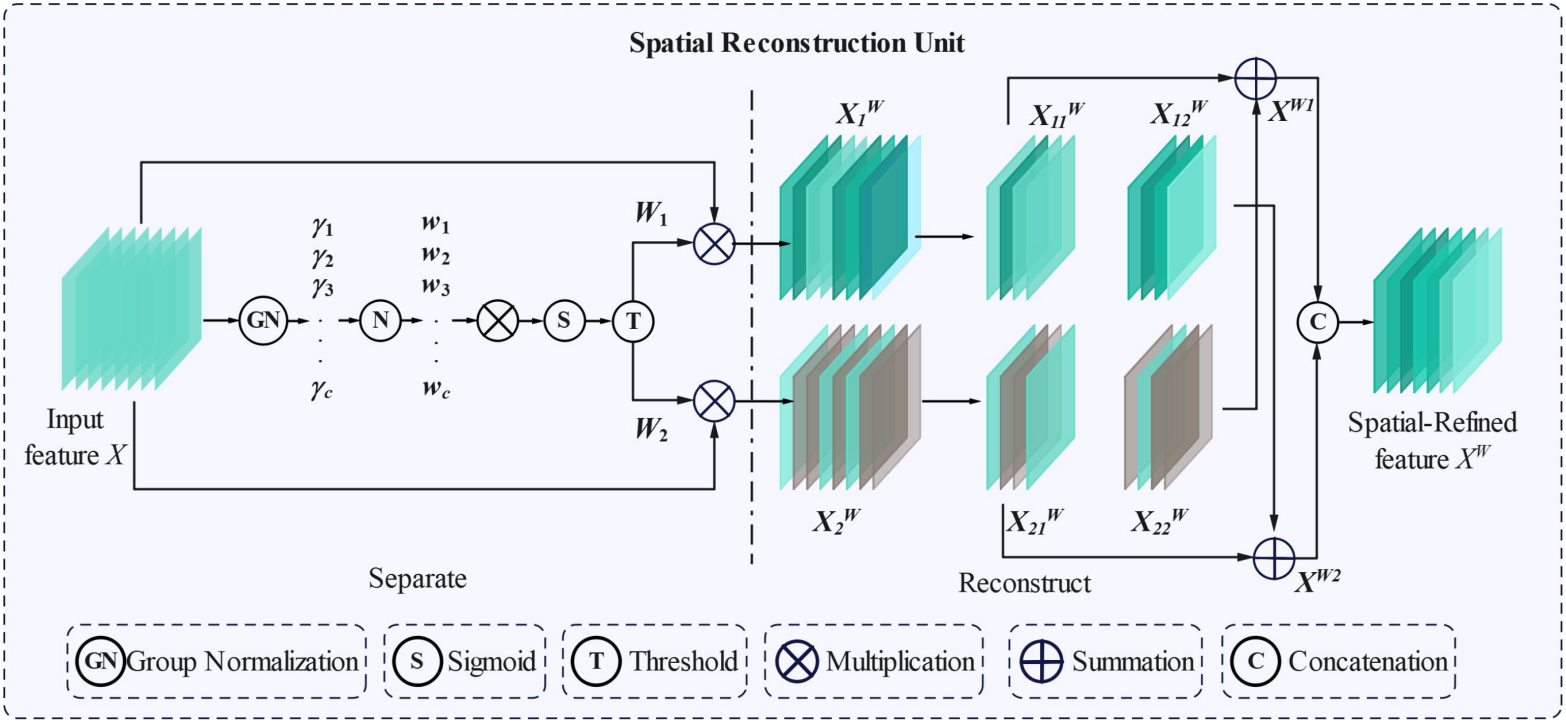
The architecture of SRCU.

The spatial reconstruction convolution unit was constructed as shown in **Figure 5**. For the input image features *X*∈*R^N^*^×^*^C^*^×^*^H^*^×^*^W^*, it is group-normalized to obtain the output feature first as shown in Equation 1. Then, parameter *γ* is introduced to measure the spatial pixel variance change after batch processing, where γ∈*R^C^*. The correlation weight *W_γ_* can be expressed as Equation 2, and the calculation process for obtaining weight *W* is shown in Equation 3.

(1) $$X_{out}=GN(X)=\gamma \frac{X-\mu }{\sqrt{\sigma^{2}+\epsilon }}+\beta$$

(2) $$W_{\gamma }=\left\{w_{i}\right\}=\frac{\gamma_{i}}{\sum_{j-1}^{C}\gamma_{i}},i,j=1,2,\cdots ,C$$

(3) $$W=Gate(Sigmoid(W_{\gamma }(X))))$$

Where *N* represents the batch axis, *C* represents the channel, and *H* and *W* are the spatial height and width of the input feature image, respectively. Here, *μ* and *σ* are the mean and standard deviation of feature *X*, respectively, where *ϵ* is a small normal number added to ensure that the denominator is not equal to zero. *γ* and *β* are defined as the affine transformation parameters of training. The two weighted features $X_{1}^{W}$ and $X_{2}^{W}$ are obtained by Multiplying the input feature *X* and the weights *W*_1_ and *W*_2_ respectively. Features $X_{1}^{W}$ contains rich information, and $X_{2}^{W}$ only has few or almost no valid features. To reduce the model computational pressure caused by spatial redundancy, a cross-reconstruction operation is performed to generate information with rich features and remove redundant information. The cross-refactoring process can be represented as Equation 4:

(4) $$\left\{ \begin{array}{l} X_{1}^{W}=W_{1}⊗X,\\ X_{2}^{W}=W_{2}⊗X,\\ X_{11}^{w}⊕X_{22}^{w}=Xw1\\ X_{21}^{w}⊕X_{22}^{w}=Xw2\\ Xw1\cup Xw2=Xw \end{array}\right.$$

After the spatial reconstruction operation of the intermediate input feature *X*, the important information features can be separated from the non-important information features, and the redundant features can be suppressed to enhance the representative features. However, spatially refined feature mapping to *X^W^* still exhibits redundancy in the channel dimension.

**Figure 6:**
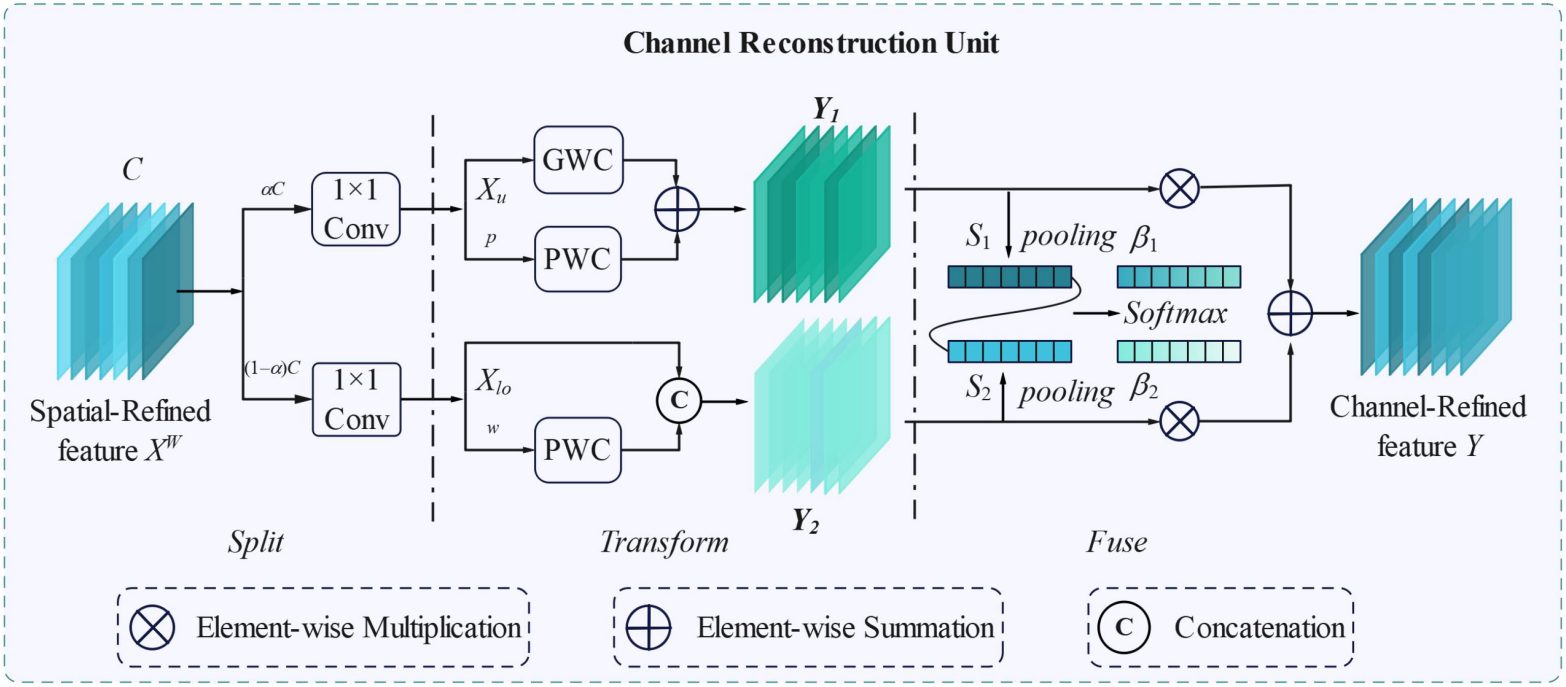
The architecture of CRCU.

To further eliminate the redundancy of channel features, the CRCU was constructed to further manipulate the intermediate features (**Figure 6**). For the input spatial refinement features *X^W^*∈*R^c^*^×^*^h^*^×^*^w^*, it is divided into upper feature *X_up_*, and lower feature *X_low_*. Efficient convolution operations (GWC and PWC) are used to replace high-cost standard convolutions to extract high-level representative information. GWC (*k* × *k*) and PWC (1 × 1) operations are performed on the same *X_up_*, and the output features are added to form a representative feature *Y*_1_, as shown in Equation 5. For the low-level feature *X_low_*, a low-cost 1 × 1 PWC operation is used to extract the feature map with shallow hidden details as a supplement to the rich-feature extractor. The upper feature *X_up_* and lower feature *X_low_* are connected to obtain the *Y*_2_ output feature map, as shown in Equation 6. The feature importance vectors *β*_1_ and *β*_2_ (Equations 7–10) are generated by the channel soft attention operation. Finally, under the guidance of the feature importance vector *β*, the channel-refined feature *Y* can be obtained by merging the upper feature *Y*_1_ and the lower feature *Y*_2_, which could be calculated by Equation 11.

(5) $$Y_{1}=M^{G}X_{up}+M^{P1}X_{up}$$

(6) $$Y_{2}=M^{P2}X_{low}\cup X_{low}$$

(7) $$S_{m}=Pooling(Y_{m})=\frac{1}{H\times W}\sum \sum Y_{c}(i,j),m=1,2$$

(8) $$\beta_{1}=\frac{e^{s1}}{e^{s1}+e^{s2}}$$

(9) $$\beta_{2}=\frac{e^{s2}}{e^{s1}+e^{s2}}$$

(10) $$\beta_{1}+\beta_{2}=1$$

(11) $$Y=\beta_{1}Y_{1}+\beta_{2}Y_{2}$$

Where, $M^{G}\in R^{\frac{\alpha c}{gr}\times k\times k\times c}$ is learnability weight matrix for GWC operations, $M^{P1}\in R^{\frac{\alpha c}{r}\times 1\times 1\times c}$ and $M^{p2}\in R^{\frac{(1-\alpha )c}{r}\times 1\times 1\times (1-\frac{1-\alpha }{r})c}$ are learnability weight matrix for PWC operations respectively. $X_{up}\in R^{\frac{\alpha c}{r}\times h\times w}$ and $Y_{1}\in R^{c\times h\times w}$ represent input feature map and the output feature map at the upper-level respectively, while $X_{low}\in R^{\frac{(1-\alpha )c}{r}\times h\times w}$ and $Y_{2}\in R^{c\times h\times w}$ represent input feature map and the output feature map at the lower-level respectively. Because the upper transformation stage uses the combined convolution of the GWC and PWC on the same feature map *X*, it can extract more representative features, and the computational cost is much lower.

**Figure 7:**
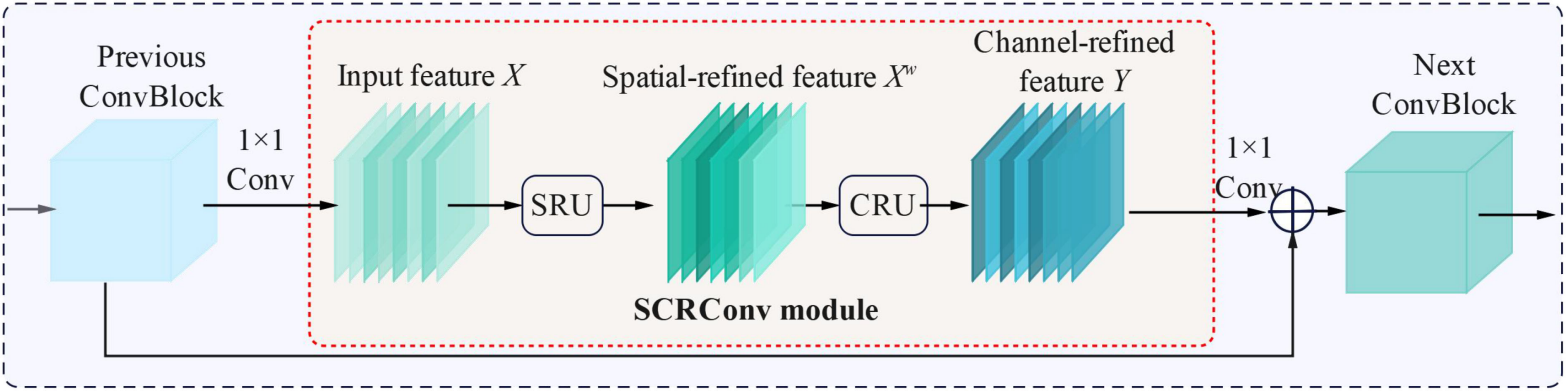
The schematic structure of SCRConv module.

By arranging the SRCU and CRCU sequentially, an efficient SCRConv module can be constructed, as shown in **Figure 7**. The core idea of the SCRConv module is to enhance the efficiency of feature representation by explicitly analyzing the spatial and channel dimension redundancy of the input feature map and conducting reconstruction, as shown in **Figure 4**. By adding the SCRConv modules to the neck parts of YOLO v8s, the redundancy of the spatial refinement feature map *X^W^*, along with the channel scale of the intermediate input feature *X*, could be reduced sharply. The representativeness of the tomato features at different ripeness levels was enhanced through low-cost operation and feature reuse, which minimized the spatial and channel redundancy of features and reduced the number of model parameters and floating-point operations.

#### 2.3.3 SimAM mechanism

**Figure 8:**
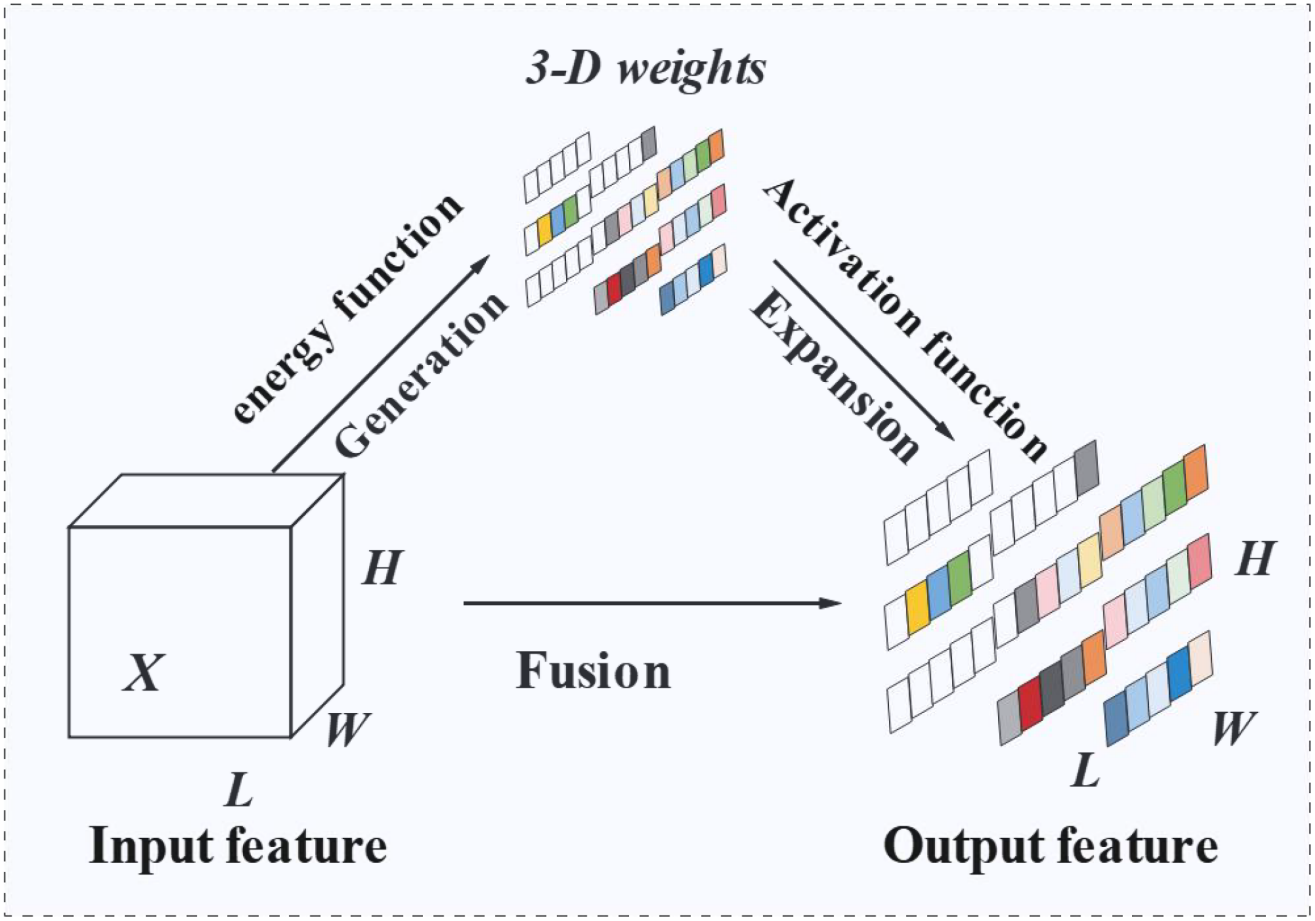
SimAM attention mechanism structure diagram.

SimAM is a parameter-free attention mechanism, which is different from the traditional channel attention generation of one weight and spatial attention generation of two weights. SimAM is an attention mechanism with three weights for attention, which can better enhance the expression ability of features in convolutional neural networks and refine the features of key regions. To achieve better attention, neurons that exhibit significant spatial inhibition should be prioritized in visual tasks. As the attention of the human brain works in tandem, it is especially important to unify the weights of the attention modules to assess the importance of each neuron. Therefore, based on neuroscience theory, an optimization energy function is proposed to measure the linear separability between neurons, and the importance of neurons is investigated. The schematic structure of the SimAM module is shown in **Figure 8**. The core idea of SimAM is based on a neuroscience theory that measures the importance of each neuron by optimizing an energy function. This process does not increase the number of network parameters but directly calculates the information difference between a neuron and its surrounding neighborhood, thereby generating joint attention weights in both spatial and channel dimensions.

The energy function is defined as Equation 12:

(12) $$e_{t}(w_{t},b_{t},y,x_{i})={\left(y_{t}-t\right)}^{2}+\frac{1}{M-1}\sum_{i=1}^{M-1}{\left(y_{O}-{\hat{x}}_{i}\right)}^{2}$$

Minimizing the above equation is equivalent to training the linear separability between neuron *T* and other neurons within the same channel. For simplicity, with binary labels and regular terms, the final energy function can be defined as Equation 13:

(13) $$e_{t}(w_{t},b_{t},y,x_{i})=\frac{1}{M-1}\sum_{i=1}^{M-1}{\left(-1-(w_{t}x_{i}+b_{t})\right)}^{2}+{\left(1-(w_{t}x_{i}+b_{t})\right)}^{2}+\lambda w_{t}^{2}$$

The analytic solution of the above equation is obtained as Equation 14:

(14) $$\left\{ \begin{array}{l} w_{t}=-\frac{2(t-\mu t)}{{\left(t-\mu t\right)}^{2}+2\sigma t^{2}+2\lambda }\\ b_{t}=-\frac{1}{2}(t+\mu t)w_{t} \end{array}\right.$$

Because all neurons on each channel follow the same distribution, the mean and variance can be calculated first for the input features in both the *H* and *W* dimensions to avoid double counting using Equation 15:

(15) $$e_{t}^{*}=\frac{4({\hat{\sigma }}^{2}+\lambda )}{{\left(t-\hat{\mu }\right)}^{2}+2{\hat{\sigma }}^{2}+2\lambda }$$

The above equation shows that the lower the energy, the greater the difference between neurons t and peripheral neurons, and the higher their importance; therefore, the importance of neurons can be obtained by 1/e*. According to the definition of the attention mechanism, the features are enhanced, and the process can be expressed as Equation 16:

(16) $$\tilde{X}=sigmoid(\frac{1}{E})⊗X$$

#### 2.3.4 Modification of bounding box regression loss function

**Figure 9:**
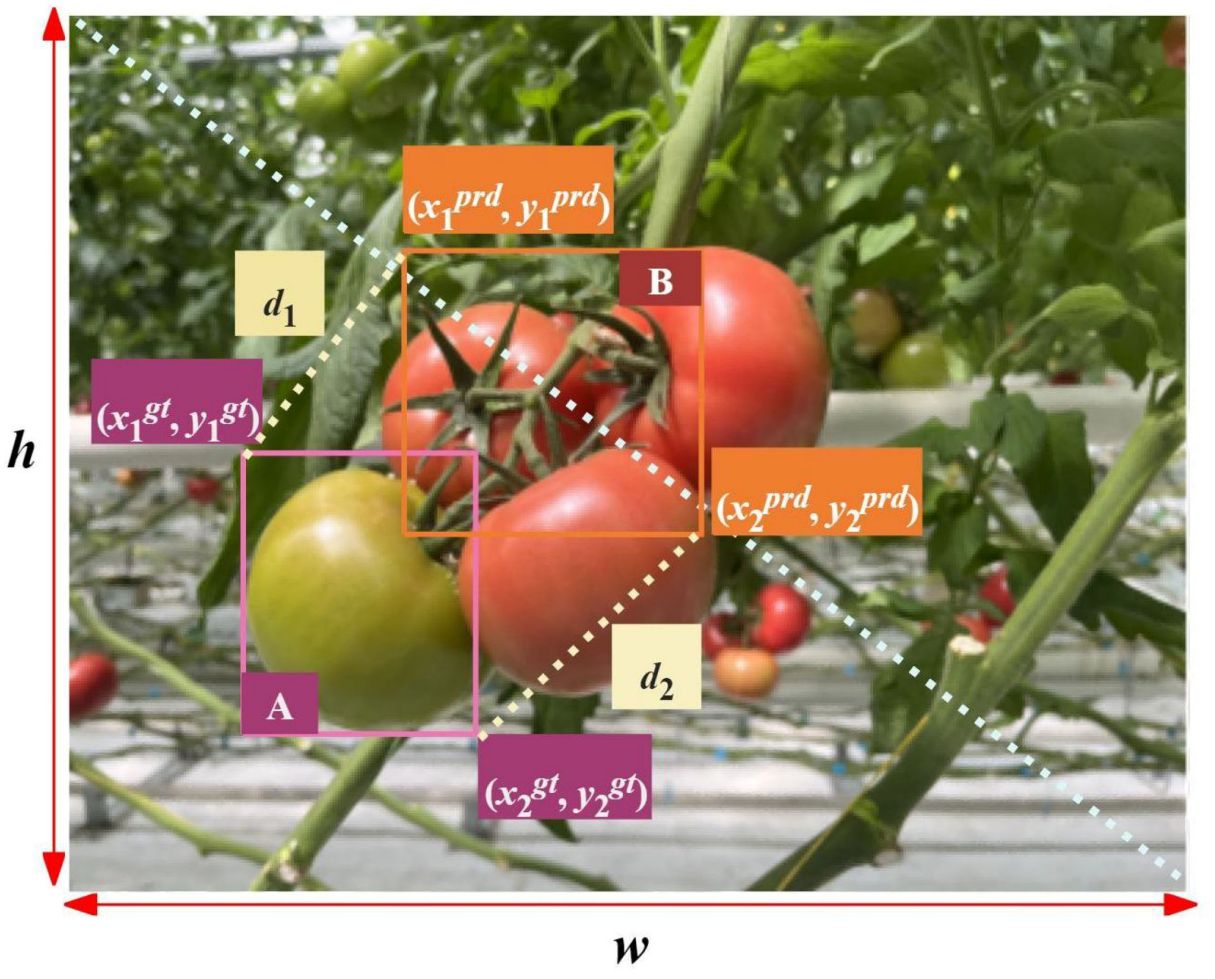
Schematic diagram of MPDIoU loss function.

Bounding box regression accuracy is crucial for the precise positioning of tomato-fruit targets. In the original YOLO v8s model, the CIoU loss function is used for the regression loss of the bounding box; however, the CIoU loss does not consider the mismatch between the direction of the predicted box and the real box. The difference between the width and height values was also ignored, which resulted in low convergence efficiency. To solve the above problems, this study proposes a new boundary box similarity comparison measure loss based on the minimum point distance (MPD). The MPDIoU loss function can explore the geometric features of a horizontal rectangular box. A schematic of the MPDIoU loss function is shown in **Figure 9**. It fully considers the size, overlap or non-overlap degree, and other complex characteristics of the target, reflecting the relative position and size relationship between tomato targets and simplifying the calculation process.

As shown in **Figure 9**, for *A* and *B* in image with the width of *w* and the height of *h*, (*x*_1_*^A^*, *y*_1_*^A^*), (*x*_2_*^A^*, *y*_2_*^A^*) denote the top-left and bottom-right point coordinates of *A*, (*x*_1_*^B^*, *y*_1_*^B^*), (*x*_2_*^B^*, *y*_2_*^B^*) denote the top-left and bottom-right point coordinates of *B*. The ground truth collection of bounding box could be described as *B_gt_* = $\left(x_{1}^{gt},y_{1}^{gt},x_{2}^{gt},y_{2}^{gt}\right)$, and the prediction collection of bounding box could be described as *B_prd =_* $\left(x_{1}^{prd},y_{1}^{prd},x_{2}^{prd},y_{2}^{prd}\right)$, where $x_{2}^{prd}>x_{1}^{prd},y_{2}^{prd}>x_{1}^{prd}$. It can be obtained as Equations 17, 18:

(17) $$d_{1}^{2}={\left(x_{1}^{prd}-x_{1}^{gt}\right)}^{2}+{\left(y_{1}^{prd}-y_{1}^{gt}\right)}^{2}$$

(18) $$d_{2}^{2}={\left(x_{2}^{prd}-x_{2}^{gt}\right)}^{2}+{\left(y_{2}^{prd}-y_{2}^{gt}\right)}^{2}$$

Then the calculation of MPDIoU and the loss function are shown as Equations 19, 20 respectively.

(19) $$MPDIoU=IoU-\frac{d_{1}^{2}}{h^{2}+w^{2}}-\frac{d_{2}^{2}}{h^{2}+w^{2}}$$

(20) $$L_{MPDIoU}=1-MPDIoU$$

The MPDIoU loss function can effectively solve the distortion of the detection frame caused by fruit overlap and reduce the missed detection of tomatoes. This could help the algorithm select the most suitable bounding box to locate the target.

## 3 Experiment and result analysis

### 3.1 Experimental environment and evaluation metrics

The experimental environment was a Windows 10 operating system. The processor was a 10th Gen Intel(R) Core i7-10700F, with a frequency of 2.90 GHz and a band memory of 32 G. The graphics card model was an NVIDIA GeForce RTX 3080 Ti. PyTorch was selected as the deep learning framework, and the development environment was CUDA 11.6 and CuDNN 8.0.5. The programming language used was Python 3.10. The development software for the entire experiment was PyCharm. To improve the detection accuracy of the model, the initial values were obtained from a pre-trained model developed by Ultralytics, which was trained on the *COCO* dataset. The training used 70% of all images. To address the memory constraints of the server, the input size of the images was adjusted to 640 × 640 pixels for all images. The initial learning rate was set to 0.0001, the number of samples per batch size was set to 32, the momentum was set to 0.938, the number of training round epochs was set to 200, the number of working multilinear processors was set to 10, and one GPU was used for acceleration (device = 0). The model was trained after the training process was defined. The test variables will also be consistent in comparative trials of different models.

In this experiment, the training and validation loss curves were plotted. Precision (*P*), recall (*R*), average precision (AP), and mean average precision (mAP) are the main metrics used to evaluate the model accuracy performance. Precision refers to the proportion of correctly predicted results among all results predicted as positive classes, whereas the essence of recall can be understood as the recall rate, that is, the proportion of positive samples in the results to all positive samples. AP is the area under the curve drawn by combining points with different precision and recall values. mAP is the value obtained by calculating the AP for all categories and then averaging the categories, which is used to reflect the overall detection effect of the model on different categories. This study focuses on the evaluation of the model’s detection capability and has a high requirement for real-time performance. In our study, mAP@0.5 was chosen as the accuracy evaluation metric, which represents the AP values of all images when the IoU was 0.5. The IoU reflects the degree of overlap between the predicted box and Ground Truth. Usually, when training VOC datasets, an IoU of 0.5 is set as the threshold. If it was greater than 0.5, it was considered correct (True); if it was less than 0.5, it was considered incorrect (False). Because mAP@0.5 uses a single loose threshold (50% overlap), which is suitable for rapid prototyping verification or general object detection scenarios, mAP@0.5 was chosen instead of mAP@0.5:0.95.

It is also necessary to examine the capacity and computational complexity of the algorithm to balance the quality of the model. Thus, the parameters, model size, FLOPs, and average detection time were used to evaluate the deployable performance. The average detection time refers to the average time required for the model to infer a single image, which is called inference time. The specific calculation process for these metrics can be found in reference (Rong J. et al., 2023).

### 3.2 Model training loss results

**Figure 10:**
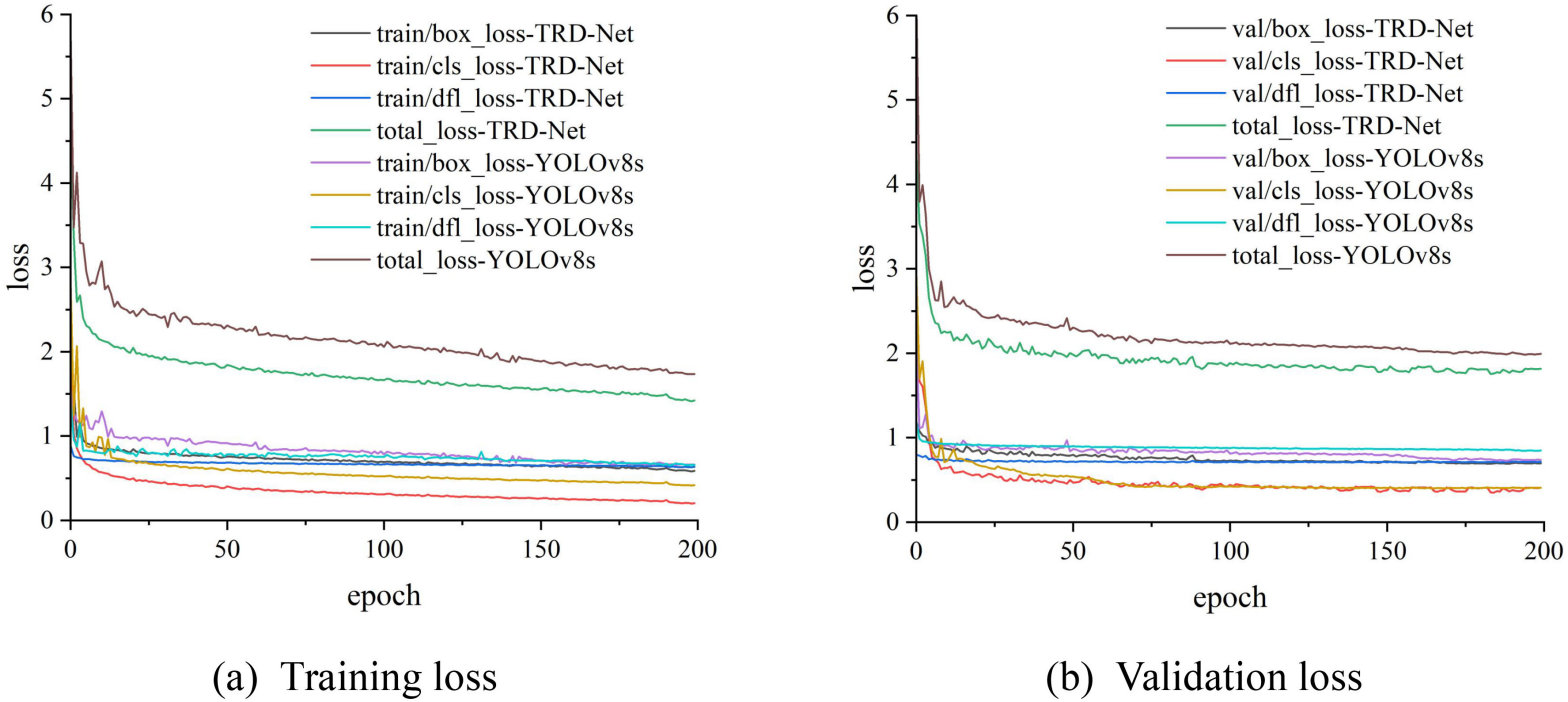
Loss curves of YOLO v8s and TRD-Net models. **(A)** Training loss. **(B)** Validation loss.

YOLO v8s and TRD-Net were trained using the same image dataset of tomatoes. To increase the interpretability of the two models and prove the effectiveness of training and evaluation, a loss function analysis was performed in this study. The loss curves associated with the bounding box, classification, and dynamic feature learning of the training and validation processing are illustrated by the exported loss data. **Figure 10** intuitively shows the loss function difference and changes in the YOLO v8s model and the proposed TRD-Net for the training and validation processes. Box_loss represents the bounding box loss weight, which is used to adjust the bounding box loss. The cls_loss represents the weight of the category loss, which is mainly used to adjust the weight of the category. The dfl_loss is a dynamic freezing loss inspired by the feature of the focal loss function, which is used to alleviate the category imbalance problem in classification identification. The total_loss is the sum of the three losses. It can be found that the convergence speed is basically the same. However, there is a significant difference between the fluctuation of the loss during training, and the loss value after convergence is also diverse. The loss value of YOLO v8s after convergence was higher than that of TRD-Net. Before improvement, the YOLO v8s model used CIoU loss as the bounding box regression function, which had a strong fitting ability during the training process. However, there is uncertainty in the calculation because the predicted box height-width ratio describes a relative value, and the high-quality and low-quality anchor frames are unfavorable to the regression loss. After improvement, the MPDIoU loss function fully considers the size, overlap or non-overlap degree, center point distance, width and height deviation, and other characteristics of the target; thus, the box_loss changes are relatively smooth, and the box_loss value is smaller. From **Figure 10**, it can be concluded that the MPDIoU loss function is better than the CIoU loss function. It is also inferred that the addition of SCRConv and SimAM may be beneficial in reducing the loss of training because of their highly effective feature extraction ability.

### 3.3 Model detection results

After training, the TRD-Net model was used to test the performance on the test set, and the detection effect was compared with the original YOLO v8s model. The test results are presented in **Table 1**. The *P* and *R* of the original YOLO v8s were 0.9268 and 0.9017, respectively. The AP of ripe and unripe tomatoes was 0.9251 and 0.9219, respectively. The AP of half-ripe tomato was only 0.8977, which is the slowest. This may be because the number of half-ripe tomatoes was low, and the feature information of half-ripe tomatoes was insufficient. The AP of TRD-Net for ripe, half-ripe, and unripe tomato detection increased by 0.0455, 0.0394, and 0.0486, respectively. When modified with SCRConv, SimAM, and MPDIoU loss, the TRD-Net significantly enhanced the representativeness of tomato features of different ripeness.

**Table 1:** Detection results for different maturity tomato using two models.

Model type	P	R	mAP@0.5	AP		
				Ripe tomato	Half-ripe tomato	Unripe tomato
YOLO v8s	0.9268	0.9017	0.9149	0.9251	0.8977	0.9219
TRD-Net	0.9671	0.9462	0.9581	0.9706	0.9371	0.9705

**Figure 11:**
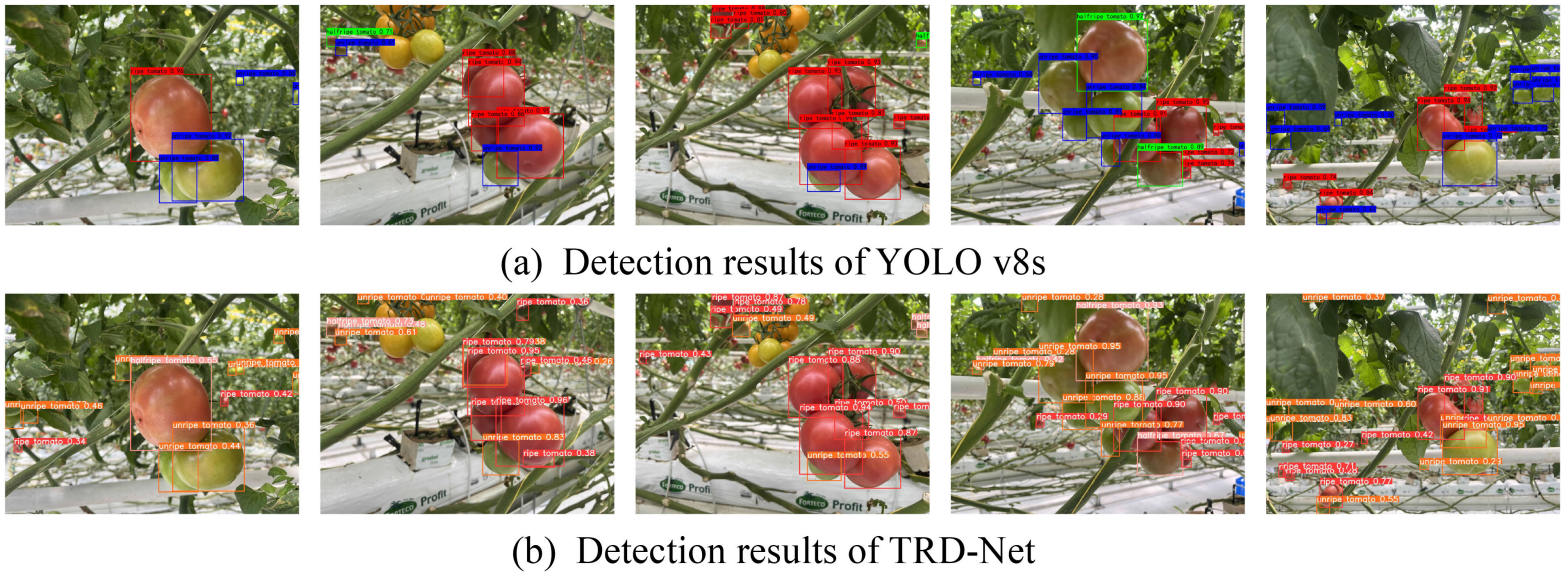
The detection results of YOLO v8s and TRD-Net. **(A)** Detection results of YOLO v8s. **(B)** Detection results of TRD-Net.

The visualization detection results of the two models are presented in **Figure 11**. The boxes of different colors indicate tomatoes with different ripeness levels. As shown in **Figure 11A**, the obvious large fruit targets can be detected and identified, whereas some small tomatoes cannot be detected. As shown in **Figure 11B**, the TRD-Net model can detect the tomato target more accurately and obtain a higher confidence level. Examples are shown in **Figure 11** also shows that the TRD-Net model enhances the detection of distant tomatoes but slightly reduces the confidence of near tomatoes. This does not affect the selective harvesting of close-range targets by the picking robot, because the confidence level remains at a relatively high value, far exceeding the set value of 0.5. The proposed model can avoid the missing detection phenomenon of the original model owing to occlusion, distance, and similar colors. Although in practical robotic harvesting, the manipulator cannot reach distant fruits, this more comprehensive detection effect is conducive to achieving automated monitoring of tomato yield and growth assessment.

**Figure 12:**
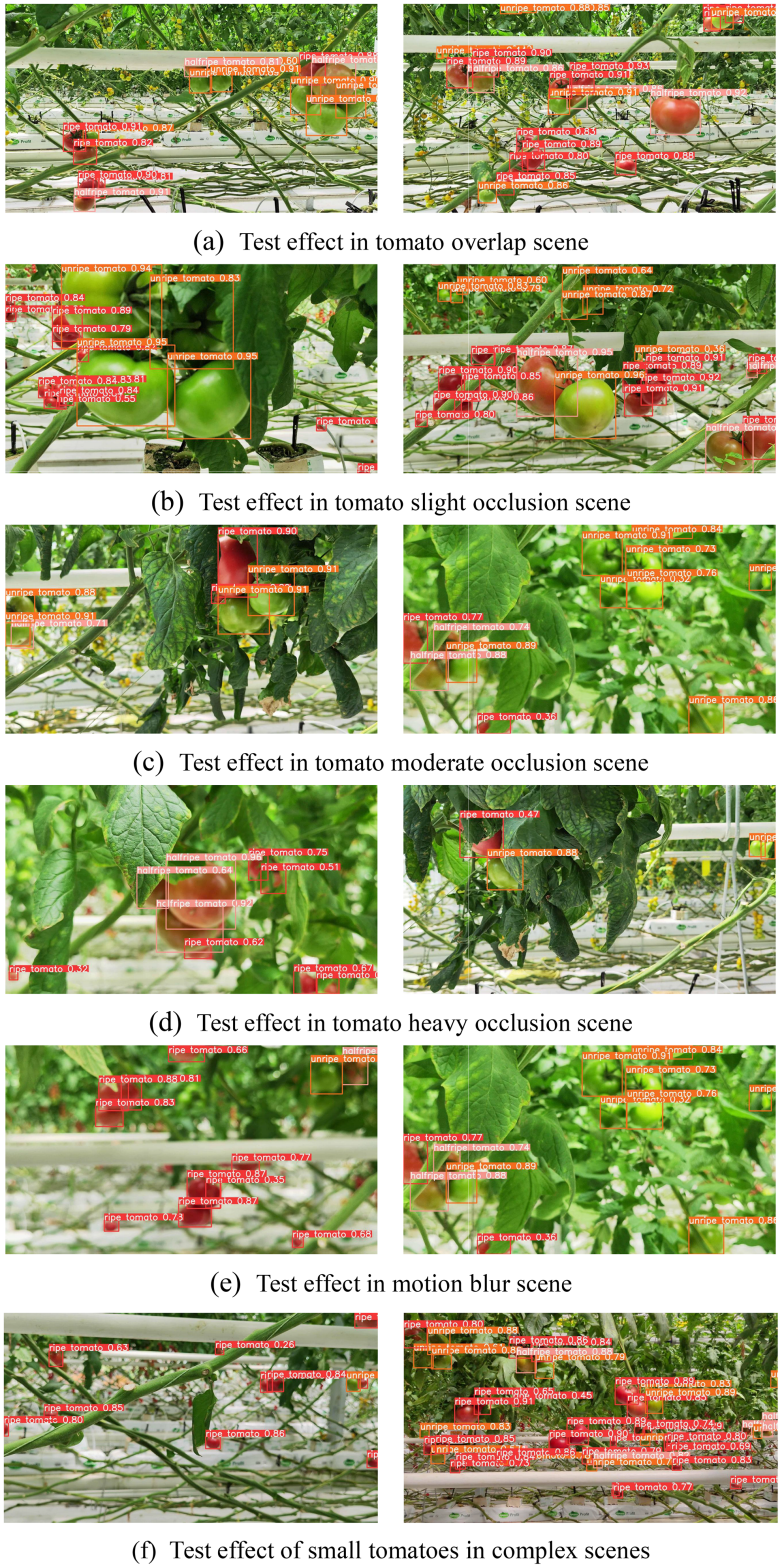
The test effect of the TRD-Net model in different scenarios. **(A)** Test effect in tomato overlap scene. **(B)** Test effect in tomato slight occlusion scene. **(C)** Test effect in tomato moderate occlusion scene. **(D)** Test effect in tomato heavy occlusion scene. **(E)** Test effect in motion blur scene. **(F)** Test effect of small tomatoes in complex scenes.

In this section, tomato images from multiple complex scenes under the same conditions are used to identify tomatoes and detect maturity levels. In the seven lines of visualization images shown in **Figure 12**, the detection results of the proposed TRD-Net model under the fruit overlap scenario, slight occlusion scene, medium occlusion scene, heavy occlusion scene, motion blur scene, and long-distance small target scene are shown from top to bottom. The red, orange, and pink boxes indicate ripe, half-ripe, and unripe tomatoes, respectively, in the visualization results. In **Figures 12A–C**, the tomatoes are overlapped and occluded, but they clearly show the detection effects of the three different ripeness tomatoes boxed and labeled with a high confidence level. The leaves obscure most of the outline of the fruit in **Figure 12D**, and the detection boxes are the outer rectangles of the existing fruit profile. However, there is a clear decrease in the confidence score. Blurs caused by rapid motion during capture are a common phenomenon that occurs frequently. The proposed model could reduce the impact of motion blur on detection, as shown in **Figure 12E**. This indicates the potential of the SCRConv module. In **Figure 12F**, there are many small targets owing to the distance. Although it is not common for harvesting robots, the vision system may face changes in the tomato scale in real scenarios. According to the detection results of multiple scenarios, the proposed method showed great robustness for different-scale tomato detection and maturity discrimination.

### 3.4 Comparison of different attention mechanisms

To verify the influence of the SimAM attention mechanism on model performance, various attention mechanism modules were added to the network structure to conduct comparative experiments. The experimental results are presented in **Table 2**. According to **Table 3**, the addition of different attention mechanisms slightly increases the model computation, and the addition of the SE, effective SE (eSE), and ECA attention modules has no positive effect on the detection accuracy of the network. Compared with other attention mechanisms, the eSE structure increases the number of parameters, model size, and computational burden of the model without contributing to the mAP. Although the CBAM and CA modules could improve the mAP@0.5 of the YOLO v8s, which increased by 0.0129 and 0.0018, respectively, the parameter amount and computation burden of the model also increased. After the SimAM attention mechanism was introduced into the network, there was no extra addition to the model parameters and computation burden, and the mAP@0.5 of the network reached 0.9387, which was increased by 0.0238 compared with the original YOLO v8s (baseline). The AP of ripe, half-ripe, and unripe tomatoes increased by 0.0231, 0.0298, and 0.0185, respectively. This indicates that the “YOLOv8s + SimAM” combination is beneficial for embedded devices. The research results of different attention mechanisms show that SimAM achieves remarkable results. When only considering the influence of the attention mechanism, adding a three-dimensional attention module has more advantages than adding a conventional channel or spatial attention module, such as CA or CBAM attention, in terms of the average detection mean average precision, and it does not bring more operational burden after adding it to the network.

**Table 2:** Experimental results of different attention mechanisms.

Attention models	Model size/M	Parameters/M	FLOPs/G	Inference time/ms	mAP@0.5	AP		
						Ripe tomato	Half-ripe tomato	Unripe tomato
YOLO v8s (baseline)	22.5	11.12	28.6	10.6	0.9149	0.9251	0.8977	0.9219
YOLO v8s + SE	22.7	11.27	28.7	10.8	0.9122	0.9243	0.8896	0.9227
YOLO v8s+CBAM	22.8	11.31	29.1	11.1	0.9278	0.9418	0.9015	0.9401
YOLO v8s + CA	22.6	11.23	28.8	10.8	0.9167	0.9276	0.8992	0.9233
YOLO v8s + ECA	22.5	11.13	28.6	10.4	0.9148	0.9250	0.8976	0.9218
YOLO v8s + eSE	24.3	13.24	29.8	11.4	0.9135	0.9247	0.8948	0.9210
YOLO v8s + SimAM	22.5	11.13	28.6	10.6	0.9387	0.9482	0.9275	0.9404

**Table 3:** Ablation test results of TRD-Net.

Model structure	P	R	mAP@0.5	Model size/M	Infer time/ms
YOLO v8s	0.9268	0.9017	0.9149	22.5	10.6
YOLO v8s + SCRConv	0.9311	0.9084	0.9185	17.6	8.7
YOLO v8s + SimAM	0.9473	0.9248	0.9387	22.5	10.5
YOLO v8s + SCRConv + SimAM	0.9613	0.9425	0.9506	17.6	8.7
YOLO v8s + SCRConv + SimAM+MPDIoU loss (TRD-Net)	0.9671	0.9462	0.9581	17.6	8.7

The Infer time means the inference time.

### 3.5 Ablation experiment of TRD-Net

Considering the characteristics of complex backgrounds and growth states, the feature extraction and attention mechanisms in YOLO v8s were proven. In this section, an ablation experiment was conducted to comprehensively evaluate the three improvements utilized in the YOLO v8s. The experimental results are presented in **Table 3**. The ablation results show that the mAP@0.5 increased by 0.0036, and the model size was reduced by 4.9 M when only the SCRConv structure was added to YOLO v8s. By introducing the SCRConv module, the redundant features of space and channels can be reduced, effective information can be better utilized, and the model size is significantly reduced. Thus, the ability and performance of the feature representation can be improved. The inference time was also reduced from 10.6 ms to 8.7 ms. When the attention mechanism is introduced to the original YOLO v8s baseline, the SimAM structure provides important three-dimensional information for the network, increases the weight of the feature region, and reduces the weight of the unimportant background features. This significantly improved the detection of tomato location and maturity. The *P*, *R*, and mAP@0.5 values increased by 0.0205, 0.0231, and 0.0238, respectively. When both the SCRConv and SimAM structures were added to YOLO v8s, the model size and inference time were the same as when only the SCRConv structure was added. The *P*, *R*, and mAP@0.5 values increased significantly. Therefore, when the loss function was improved to replace the CIOU loss, the MPDIoU loss function effectively solved the distortion of the detection frame caused by fruit overlap and effectively reduced the problem of missed detection of tomato fruits. At this time, the model performance was the highest, with an mAP@0.5 of 0.9581, and the model size and inference time remained at 17.6 M and 8.7 ms, respectively.

### 3.6 Comparison with the SOTA models

To further analyze the effectiveness of the proposed model in the implementation of tomato recognition and ripeness detection tasks, we selected the most advanced object detection algorithms for comparison. The TRD-Net model was compared with the YOLO v4, YOLO v5, YOLO v6, YOLO v7, and original YOLO v8 serial models. The model training environment and datasets were consistent during the experiments. **Table 4** shows the comparison results of different models under the premise that the input image size is uniformly 640 × 640 pixels. The mAP@0.5 of YOLO v4 was the lowest, below 0.8. As the version evolution of YOLO, it can be concluded that the mAP@0.5 is basically increased, but the deeper network structure limits the increase in mAP@0.5. The YOLO v8n has the smallest model size and parameters, but its mAP@0.5 is lower than that of YOLO v8s. This is also the main reason for choosing YOLO v8s as the baseline for the study. The TRD-Net achieved the highest mAP@0.5 and fastest inference speed. Compared with the original YOLO v8s and other models, the proposed TRD-Net model achieved a mAP of 0.9581, which is the highest result. The inference time was the lowest, which reflects the fastest detection speed among all models. This is because the SCRConv structure designed in this study offers an effective feature extraction ability, and the SimAM attention mechanism also improves the feature representation effects. Although the model size was larger than that of YOLO v8n, the inference time was lower. This indicates that the improved model has a higher processing efficiency for feature maps. Thus, the ease of use of the model on mobile devices has been greatly improved. The TRD-Net achieved the best balance between accuracy and real-time performance. In summary, the proposed TRD-Net model diverges from state-of-the-art models in the same experimental environment, which is notably promising for the visual perception of selective harvesting robots.

**Table 5:** The comparison results of different models.

Model types	mAP@0.5	Model size/M	Parameters/M	FLOPs/G	Inference time/ms
YOLO v4	0.7965	491.7	64.37	271.1	53.4
YOLO v5s	0.8217	14.3	7.26	16.3	18.4
YOLO v5m	0.8236	40.5	21.52	49.1	29.3
YOLO v5l	0.8329	93.7	47.73	109.6	42.5
YOLOv6-tiny	0.8277	20.5	15.43	36.9	22.9
YOLO v7	0.8413	73.6	37.86	106.2	29.7
YOLO v8n	0.8772	6.2	3.01	8.9	20.1
YOLO v8s	0.9149	22.5	11.12	28.6	40.2
YOLO v8m	0.9275	52.1	25.84	79.1	59.1
YOLO v8l	0.9093	87.6	43.61	165.3	69.2
YOLO v10m	0.9335	25.9	15.4	51.9	12.2
TRD-Net	0.9581	17.6	8.93	22.3	8.7

### 3.7 Heat map visualization interpretation

To verify how the proposed model focused on different tomatoes during fruit identification and detection, Grad-CAM technology was applied to interpret the model’s detection process subjectively and visually. The principle of Grad-CAM is based on the following Equations 21, 22:

(21) LGrad−CAMc=ReLU(∑kαkcAc)

(22) $$\alpha_{k}^{c}=\frac{1}{Z}\sum_{i}\sum_{j}\frac{\partial y^{c}}{\partial A_{ij}^{k}}$$

**Figure 13:**
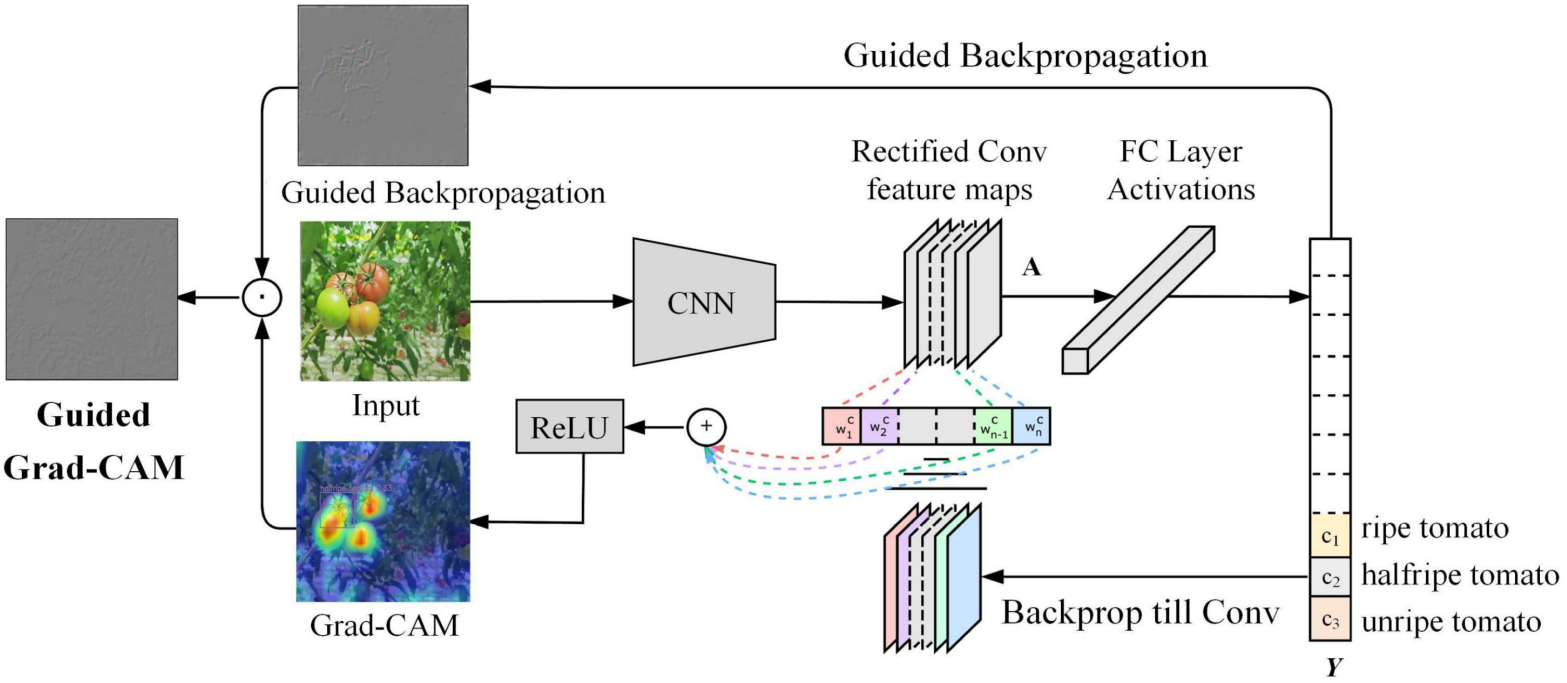
The generation principle of Grad-CAM heat map.

Where, *Z* is the number of pixels in the feature map, *y^c^* is the score corresponding to category *c*, $A_{ij}^{k}$ denotes the pixel value at position(*i*, *j*) in the *k^th^* feature map, and $\alpha_{k}^{c}$ is the weight of the *k*th feature map for category *c*. The generation principle of heat map is shown in **Figure 13**.

**Figure 14:**
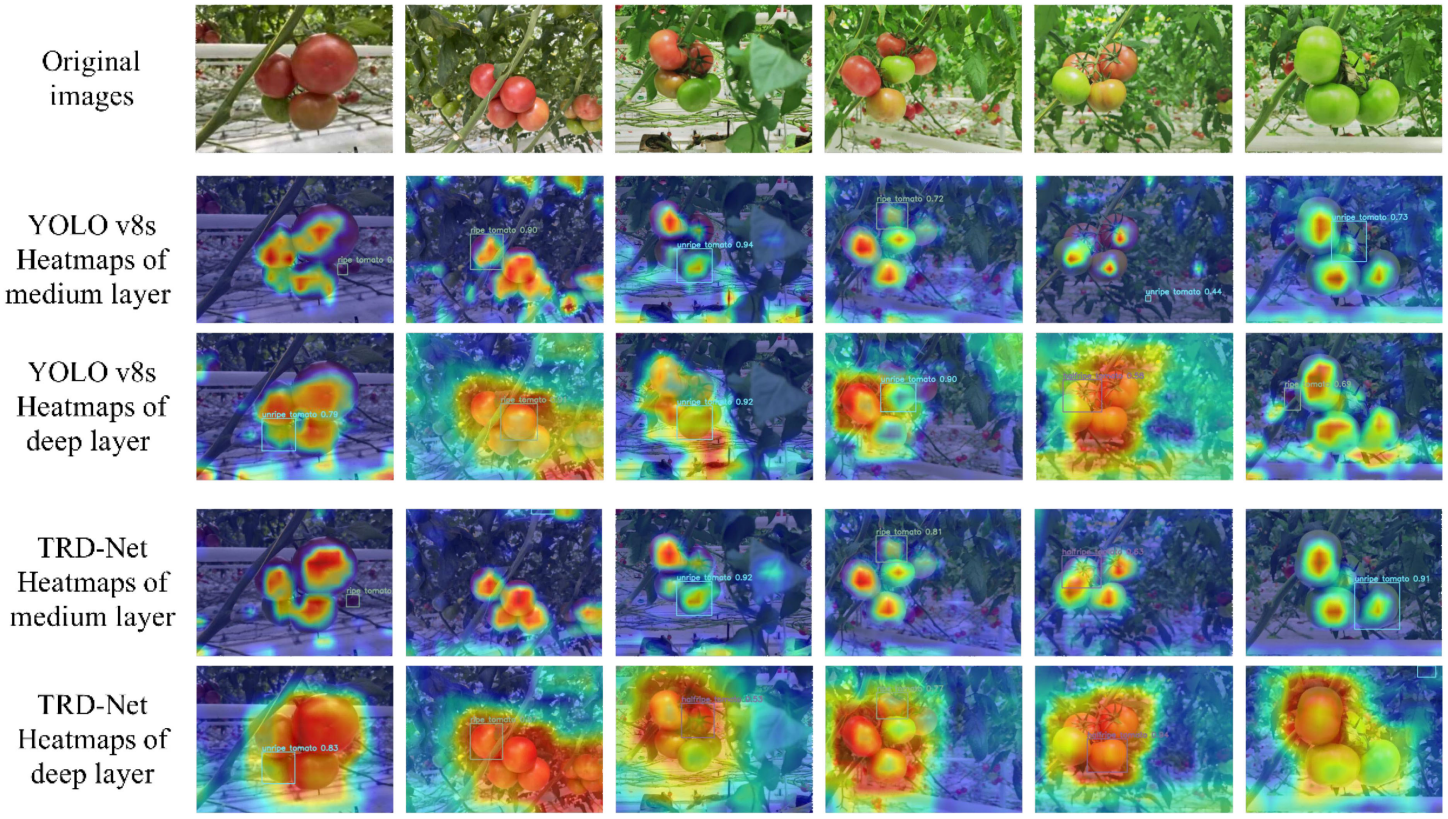
Heatmaps for different layers activation visualizations of YOLO v8s and TRD-Net.

In this section, the medium- and deep-layer heatmaps of the YOLO v8s and TRD-Net models are illustrated in **Figure 14**. The heat maps effectively show the degree of responsiveness in tomato recognition and ripeness detection, with areas that contribute more to the model detection shown in red and areas that contribute less shown in blue. From the visualization results, it can be found that the YOLO v8s and TRD-Net models could focus well on the tomato fruits. However, when the YOLO v8s model detects fruits, there was an object offset, and it still had a relatively high level of attention at the positions of non-fruit targets. The proposed TRD-Net model can improve the extraction performance of fruit target features while inhibiting the extraction of background features. This can effectively solve the problem of accurate detection of tomatoes in complex environments. The proposed model pays good attention to the area where the tomato fruits are located in the images and extracts stronger target features from images with weak semantics. The visualization results further show that as the number of layers of the model increases from shallow to deep, the response area of the model attention to tomato gradually increases. This phenomenon indicates that the model can cope well with the identification needs of targets at different scales, showing robustness and generalization ability.

## 4 Discussion

The purpose of this study is to deploy and apply the technology of fruit ripeness detection to agricultural intelligent equipment to meet the task of selective tomato harvesting in complex gardens. A TRD-Net model for real-time tomato target recognition and ripeness discrimination was proposed, and the high performance of the TRD-Net using images captured in complex horticulture scenarios was demonstrated. The results showed that the vast majority of tomatoes were detected, and the ripeness classification was accurate. The introduction of SCRConv enhanced the representativeness of tomato features of different ripeness through low-cost operation and feature reuse. It also reduces the storage space and computational cost of the YOLO v8s model by reducing the spatial and channel redundancy between features in the convolutional neural network while improving the accuracy and generalization for tomato detection tasks. Compared with the attention module of some attention models, which only focus on channels or spaces, the SimAM used in this study can obtain three-dimensional weight information by mining the importance of neurons, so that the detection network can better focus on the tomato fruit target to be detected. Under the comprehensive measurement of various accuracy and parameter quantities, the 3D attention improvement effect is more obvious, thereby effectively improving the feature extraction ability of the network. The modification of the loss function also contributes to the model precision performance, because the MPDIoU loss function fully considers the size, overlap or non-overlap degree, center point distance, width and height deviation, and other characteristics of tomatoes in a complex environment. Experimental results demonstrate that the proposed TRD-Net model significantly outperforms existing object detection models, delivering superior accuracy with relatively fewer parameters and fewer FLOPs.

Current research indicates that scholars have made progress in using deep learning and machine vision to detect fruit in plants, highlighting the rapid development of this field. Noteworthy examples include research on apple detection (Ma et al., 2024; Wen et al., 2024; Zhang et al., 2025) and tomato identification (Chen et al., 2024a; Chen et al., 2024b). However, research on tomato detection for selective harvesting remains relatively scarce and has certain limitations. For instance, Gu et al. (2024) constructed an improved RTDETR-CASA model for rapid tomato fruit detection. The FPS of RTDE-CASA is relatively high, whereas the mAP@0.5 is only 0.86, which is significantly lower than 0.9587 in this study. Wang et al. (2024) improved the YOLO v8s model to detect tomato fruits at different ripeness stages, and this research did not consider the influence of fruit occlusion. Their research results showed that the mAP@0.5 reached 0.914 and the FLOPs reached 42.4 G. In contrast, this study examined a more intricate growth scenario in the environment of a tomato garden. The developed TRD-Net model demonstrated superior generalization capability and robustness when confronted with overlapped, occluded, and blurred mixed interference, and variations in scale. It should be noted that the current model was trained and evaluated primarily under relatively homogeneous greenhouse conditions using specific tomato varieties. Although the proposed enhancements were designed to improve robustness, the generalizability of the proposed method to substantially different environments warrants further validation and potential domain adaptation in future studies.

## 5 Conclusion

Differences in the ripeness of tomatoes on the same plant have led to new requirements for time-based selective harvesting of tomatoes. Simultaneously, the shelf life of tomato fruits is relatively short, with approximately 30% of the fruit lost before and after harvest due to the significant softness of ripe tomatoes. Therefore, it is of great economic significance to use vision technology to realize the online detection of tomato ripeness before harvest. The new generation fruit detection model requires higher accuracy, lower computational overhead, faster speed, and easier deployment. To meet the above requirements, a meticulously crafted tomato image dataset was constructed for multitask classification. The one-stage anchor-free YOLO v8s model was optimized to avoid feature redundancy in the tomato detection process, thus shortening the detection time, which can be widely applied to other agricultural fields. The performance of the proposed TRD-Net is robust in complex environments involving fruit occlusion, fruit overlap, and motion blur. The proposed TRD-Net is more concise in design, has fewer requirements for memory and calculation, and realizes the prediction of higher probability values, which can meet real-time gardening detection tasks and present more comprehensive and efficient results in complex environments. This study provides a marked contribution to the establishment of high-performance robust models and provides theoretical support and practical reference for the further development of smart agriculture.

Although this study presents an efficient model for detecting tomato ripeness, it is not without limitations. The influence of tomato variety also necessitates further investigation. In future work, we will focus on developing a more diverse dataset covering different tomato varieties. By enlarging the dataset, the model can be used for the detection of various fruits. From a deployment perspective, the reduced computational cost of the TRD-Net (6.3 GFLOPs) makes it a more feasible candidate for edge devices than the baseline. However, achieving real-time performance on very low-end hardware systems may require additional optimization techniques such as model quantization or pruning. Efforts will be directed towards developing an end-to-end, efficient, and low-overhead ripeness detection system to improve the model transplantation performance of the edge mobile platform. From an industrial perspective, a set of automatic monitoring equipment for tomatoes can be designed to facilitate rapid detection and monitoring. Combining other sensors, such as depth cameras, for spatial measurements can further reduce the dependence on single RGB images. Future work will focus on implementing and testing these optimizations to facilitate practical deployment in resource-constrained harvesting robot. Ultimately, the proposed model will serve as a crucial “eye” and “brain” for planting managers, enabling them to implement selective harvesting using advanced robotic equipment.

## Data Availability

The raw data supporting the conclusions of this article will be made available by the authors, without undue reservation.
